# The Rev1 interacting region (RIR) motif in the scaffold protein XRCC1 mediates a low-affinity interaction with polynucleotide kinase/phosphatase (PNKP) during DNA single-strand break repair

**DOI:** 10.1074/jbc.M117.806638

**Published:** 2017-08-16

**Authors:** Claire Breslin, Rajam S. Mani, Mesfin Fanta, Nicolas Hoch, Michael Weinfeld, Keith W. Caldecott

**Affiliations:** From the ‡ Genome Damage and Stability Centre, School of Life Sciences, University of Sussex, Science Park Road, Falmer, Brighton BN19RQ, United Kingdom,; the § Department of Experimental Oncology, Cross Cancer Institute, Edmonton, Alberta T6G 1Z2, Canada, and; the ¶ CAPES Foundation, Ministry of Education of Brazil, Brasilia/DF 70040-020, Brazil

**Keywords:** base excision repair (BER), DNA damage, DNA damage response, DNA repair, oxidative stress

## Abstract

The scaffold protein X-ray repair cross-complementing 1 (XRCC1) interacts with multiple enzymes involved in DNA base excision repair and single-strand break repair (SSBR) and is important for genetic integrity and normal neurological function. One of the most important interactions of XRCC1 is that with polynucleotide kinase/phosphatase (PNKP), a dual-function DNA kinase/phosphatase that processes damaged DNA termini and that, if mutated, results in ataxia with oculomotor apraxia 4 (AOA4) and microcephaly with early-onset seizures and developmental delay (MCSZ). XRCC1 and PNKP interact via a high-affinity phosphorylation-dependent interaction site in XRCC1 and a forkhead-associated domain in PNKP. Here, we identified using biochemical and biophysical approaches a second PNKP interaction site in XRCC1 that binds PNKP with lower affinity and independently of XRCC1 phosphorylation. However, this interaction nevertheless stimulated PNKP activity and promoted SSBR and cell survival. The low-affinity interaction site required the highly conserved Rev1-interacting region (RIR) motif in XRCC1 and included three critical and evolutionarily invariant phenylalanine residues. We propose a bipartite interaction model in which the previously identified high-affinity interaction acts as a molecular tether, holding XRCC1 and PNKP together and thereby promoting the low-affinity interaction identified here, which then stimulates PNKP directly.

## Introduction

XRCC1 is a molecular scaffold protein that interacts with multiple components of the single-strand break repair (SSBR)^4^ pathway including DNA polymerase β (Pol β) (1, 2), polynucleotide kinase/phosphatase (PNKP) (3, 4), Aprataxin (APTX) (5, 6), Aprataxin- and PNKP-like factor (7–9), and DNA ligase 3α (Lig3α) (10, 11). XRCC1 also interacts with poly(ADP-ribose), the product of PARP1 and/or PARP2 activity, via its central BRCT1 domain, thereby enabling its accumulation at chromosomal SSBs (12–16). The interactions mediated by XRCC1 affect protein partners in several ways including stabilization, recruitment to sites of SSBs, and in some cases enzymatic stimulation. One of the most important interactions mediated by XRCC1 is with PNKP, because many of the DNA strand breaks arising in cells are substrates for this enzyme (17–21). PNKP possesses both DNA 5′-kinase and DNA 3′-phosphatase activities, which together convert 5′-hydroxyl and 3′-phosphate termini to canonical 5′-phosphate and 3′-hydroxyl moieties, respectively, thereby enabling the final steps of DNA gap filling and DNA ligation (22–24).

The importance of PNKP is illustrated by the observation that mutations in this gene result in the hereditary neurological diseases *microcephaly with early onset seizures* (MCSZ) and *ataxia oculomotor apraxia 4* (AOA4) (25–27). MCSZ is characterized by neurodevelopment defects and reduced cerebellar size, whereas AOA4 is characterized by progressive cerebellar degeneration and ataxia. It is unclear why mutations in the same protein can result in two different neurological diseases but this may reflect the varying impact of the different mutations on PNKP function and/or the additional role of this protein in non-homologous end joining. None of the mutations, to date, result in the complete absence of enzyme activity, suggesting that PNKP may be essential for viability (28). Consistent with this idea, germ line deletion of PNKP is lethal in mouse (29).

XRCC1 interacts with PNKP by both phosphorylation-dependent and phosphorylation-independent mechanisms (3, 4, 30). The phosphorylation-dependent interaction is mediated by a forkhead-associated (FHA) domain in PNKP and a cluster of three CK2 phosphorylation sites at Ser^518^/Thr^519^/Thr^523^ in XRCC1, resulting in a very high-affinity interaction with full-length phosphorylated XRCC1 (*K_d_* ∼ 3.5 nm) (4, 30, 31). XRCC1 stimulates PNKP activity at limiting concentrations of the latter (3, 4), in part at least by displacing PNKP from its substrate and thereby releasing the enzyme for additional cycles of activity (30, 32). However, whereas the phosphorylation-dependent interaction contributes to PNKP stimulation it is not essential for this purpose, because non-phosphorylated XRCC1 can also stimulate PNKP to a lesser extent (3, 32, 33). Consistent with this, full-length XRCC1 contains a second binding site that mediates a lower affinity, phosphorylation-independent, interaction (*K_d_* ∼ 30 nm) with the catalytic domain of PNKP (3, 32, 33). However, the location and physiological importance of this binding site in XRCC1 are unknown. Given the importance of XRCC1 and PNKP for normal neurological function in mouse and man (25, 29, 34) we have identified in this work the location and importance of the low-affinity binding site in XRCC1.

## Results

**Figure 1.**
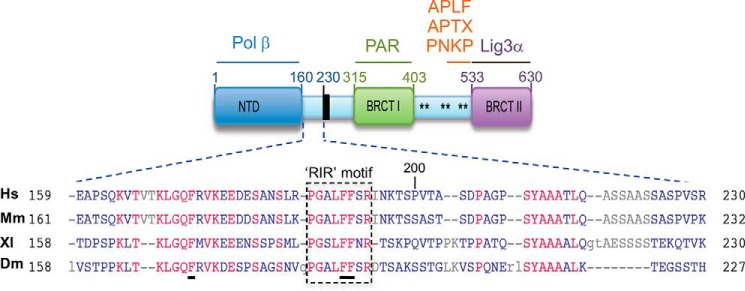
**A novel conserved domain in XRCC1.** Shown is a schematic of established molecular interaction sites in human XRCC1. A highly conserved domain of ∼40 amino acids located between the NTD and the NLS and containing three invariant phenylalanine residues (*underlined*) including a putative RIR motif (*boxed*), is highlighted. *Hs, Homo sapiens*; *Mm*, *Mus musculus*; *XL*, *Xenopus laevis*; *Dm*, *Drosophila melanogaster*.

**Figure 2.**
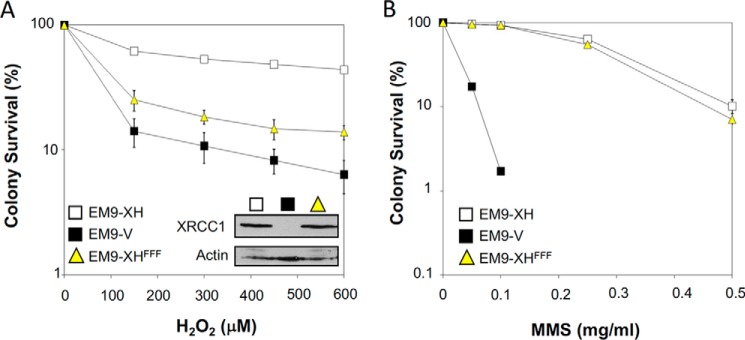
**The RIR motif is important for XRCC1-dependent cell survival following oxidative stress.** *A*, clonogenic survival of *XRCC1*-mutant EM9 cells stably transfected with empty pCD2E vector (*V*) or with pCD2E expression constructs encoding full-length wild-type XRCC1-His (*EM9-XH*) or XRCC1-His^FFF^ (*EM9-XH^FFF^*). Cells were treated with the indicated concentrations of H_2_O_2_ for 15 min and then in drug-free medium for 10–14 days to allow colony formation. Data are the mean ± S.E. of three independent experiments. Where not visible, *error bars* are smaller than the symbols. *B*, clonogenic cell survival following MMS treatment, as above.

**Figure 3.**
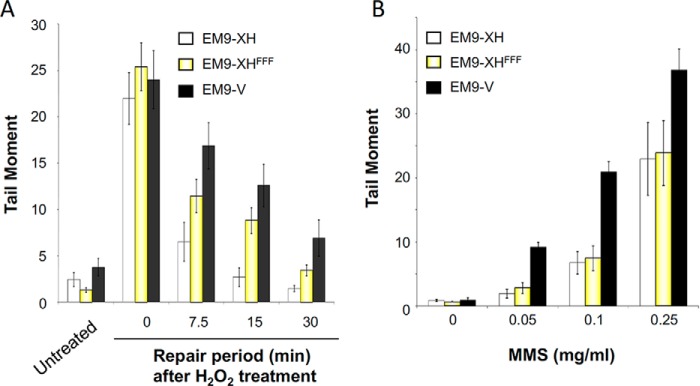
**The RIR motif is important for XRCC1-dependent SSBR following oxidative stress.** *A*, chromosomal SSBR rates were measured in the above EM9 cell lines in alkaline comet assays following treatment with 150 μm H_2_O_2_ for 20 min on ice, followed by recovery in drug-free medium for the indicated time at 37 °C. *B*, steady state SSB levels as an indicator of chromosomal SSBR rates were measured in the above EM9 cell lines in alkaline comet assays following treatment with the indicated concentration of MMS for 15 min at 37 °C. Data are the mean ± S.E. of three independent experiments.

XRCC1 is a scaffold protein comprised of multiple molecular interaction domains (Fig. 1*A*). Although binding partners for most of these domains have been identified, XRCC1 also possesses a poorly characterized but highly conserved region denoted the “Rev1-interacting region” (RIR) located just upstream of the BRCT1 domain (35). Of particular interest within this region is a cluster of conserved residues that includes the invariant phenylalanine residues Phe^173^, Phe^191^, and Phe^192^ (Fig. 1). To examine the importance of this region, we mutated the three conserved phenylalanine residues to alanine and examined the mutated protein (denoted XRCC1-His^FFF^) for ability to complement *XRCC1*-mutant EM9 Chinese hamster ovary (CHO) cells. XRCC1-His^FFF^ was unable to restore normal levels of cell survival in EM9 cells following treatment with H_2_O_2_ (Fig. 2*A*). However, this was not the case following methyl methanesulfonate (MMS) treatment, to which EM9 cells expressing either XRCC1-His or XRCC1-His^FFF^ exhibited similar levels of sensitivity over the concentration range employed (Fig. 2*B*). In agreement with these data, XRCC1-His^FFF^ was less able to support normal rates of SSBR than XRCC1-His following H_2_O_2_ treatment (Fig. 3*A*), but was equally able to support normal rates of base excision repair following treatment with MMS (Fig. 3*B*). The ability of the mutated protein to complement phenotypes associated with MMS-induced damage, but not H_2_O_2_-induced damage, suggests that this mutation does not disrupt general folding of the protein. Rather these data suggest that the triple phenylalanine mutation is a separation-of-function mutation that impedes the repair of DNA strand breaks induced at sites of DNA oxidation but not those induced at sites of DNA alkylation.

**Figure 4.**
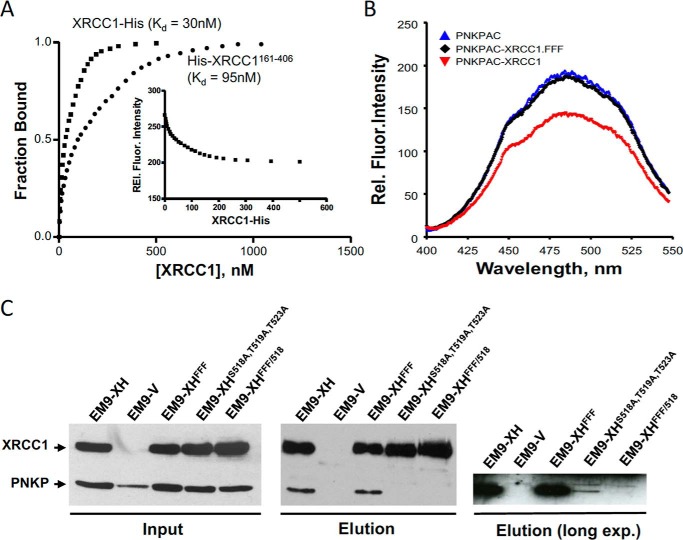
**The RIR motif mediates the phosphorylation-independent interaction with PNKP.** *A*, phosphorylation-independent interaction of PNKP with full-length and truncated XRCC1. Acrylodan-labeled PNKP was excited at 380 nm, the relative fluorescence intensities were monitored at 485 nm (the data for XRCC1-His is provided in the *inset*) as a function of full-length XRCC1-His (■) or truncated His-XRCC1^161–406^ (●). The concentrations of PNKP-AC used were 20 and 80 nm with full-length XRCC1, and truncated His-XRCC1^161–406^, respectively. A representative plot of the fraction of PNKP-AC bound *versus* XRCC1 concentration is shown. The *K_d_* values reported represents the mean ± S.E. (*n* = 3). *B*, mutation of the phenylalanine motif prevents phosphorylation-independent interaction of XRCC1 with PNKP, *in vitro*. PNKP-AC (80 nm) was excited at 380 nm, and the emission fluorescence was measured between 400 and 550 nm. Note that the emission peak was ∼490 nm and was quenched only by wild-type XRCC1 and not by His-XRCC1^FFF^ mutant. Data are based on three experiments using varied concentrations of the triple mutant. The scans shown represent data for a 1:1 molar ratio for PNKP to wild-type XRCC1 and a 1:6 molar ratio of PNKP to XRCC1^FFF^. *C*, cell extract from EM9 cells transiently transfected with pcD2E-PNKP and either empty pcD2E vector (*EM9-V*) or a pCD2E expression construct encoding full-length XRCC1-His (*EM9-XH*), XRCC1-His^FFF^ (*EM9-XH^FFF^*), XRCC1-His^S518A/T519A/T523A^ (*EM9-XH^S518A/T519A/T523A^*), or XRCC1-His^FFF/S518A/T519A/T523A^ (*EM9-XH^FFF/518^*) was incubated with nickel-nitrilotriacetic acid-agarose and histidine-tagged protein complexes recovered as described under “Experimental procedures.” Aliquots of the column input and eluate were fractionated by SDS-PAGE and immunoblotted with anti-XRCC1 and anti-PNKP antibody. The gel on the *right* is an overexposure of the PNKP blot in the eluate sample.

One possible explanation for the selective impact of the phenylalanine mutations is that they disrupt the interaction of XRCC1 with PNKP. This is because although PNKP is predicted to play a minor role in the repair of MMS-induced DNA breaks, it is predicted to play a major role in the repair of oxidative DNA breaks (20). The high-affinity interaction of XRCC1 with PNKP is mediated by the phosphorylated CK2 sites located at Ser^518^/Thr^519^/Thr^523^, but the site of the low-affinity interaction is unclear. The latter interaction is far weaker than the phosphorylation-dependent interaction but is nevertheless important for PNKP stimulation (3, 4, 30). To examine whether the phenylalanine motif might mediate the phosphorylation-independent interaction we examined recombinant histidine-tagged XRCC1 purified from *Escherichia coli* for interaction with an enzymatically active derivative of PNKP labeled with acrylodan (denoted PNKP-AC) in fluorescence quenching experiments. For these experiments we employed full-length XRCC1-His and His-XRCC1^161–406^, a truncated derivative of XRCC1 encoding the central 245 amino acids spanning the phenylalanine motif. Full-length XRCC1-His resulted in partial quenching of AC fluorescence at 490 nm in a concentration-dependent manner (Fig. 4*A*). Nonlinear regression analysis of this binding data revealed unimodal binding with a *K_d_* ∼ 30 nm for XRCC1-His, consistent with our previous report (32), and *K_d_* ∼ 95 nm for His-XRCC1^161–406^, indicating ∼3-fold weaker but nevertheless tight binding by the latter (Fig. 4*A*). Importantly, mutation of the three phenylalanine residues in full-length XRCC1-His (His-XRCC1^FFF^) induced only ∼4% 6-acryloyl-2-dimethylaminonaphthalene (AC) quenching when added in 6-fold excess (Fig. 4*B*). This level of quenching is too low to obtain a *K_d_*, suggesting that the phenylalanine motif is required for the low-affinity interaction with PNKP-AC.

To examine the phenylalanine motif for interaction with PNKP in cells we co-transfected EM9 cells with constructs encoding human PNKP and either wild-type full-length XRCC1-His or mutant full-length XRCC1-His^FFF^, and affinity purified His-tagged protein complexes by metal-chelate chromatography (Fig. 4*C*). Similar amounts of PNKP co-purified with either XRCC1-His protein, suggesting that mutation of the phenylalanine motif did not greatly reduce XRCC1 interaction with PNKP. This was not surprising, because cellular XRCC1 is constitutively phosphorylated by CK2 and thus can bind PNKP via the high-affinity interaction. Indeed, as reported previously (4, 18), mutation of the CK2 phosphorylation sites that mediate the high-affinity interaction greatly reduced PNKP co-precipitation (Fig. 4*C, XRCC1-His^S518A/T519A/T523A^*). Importantly, however, overexposure of the autoradiograph revealed a small amount of residual co-purified hPNKP, which was further reduced or ablated by additional mutation of the phenylalanine motif (Fig. 4*C*, *right*). Together, these data suggest that whereas the PNKP interaction is determined primarily by the high-affinity phosphorylation-dependent interaction, the low-affinity interaction is disrupted by mutation of the phenylalanine motif.

**Figure 5.**
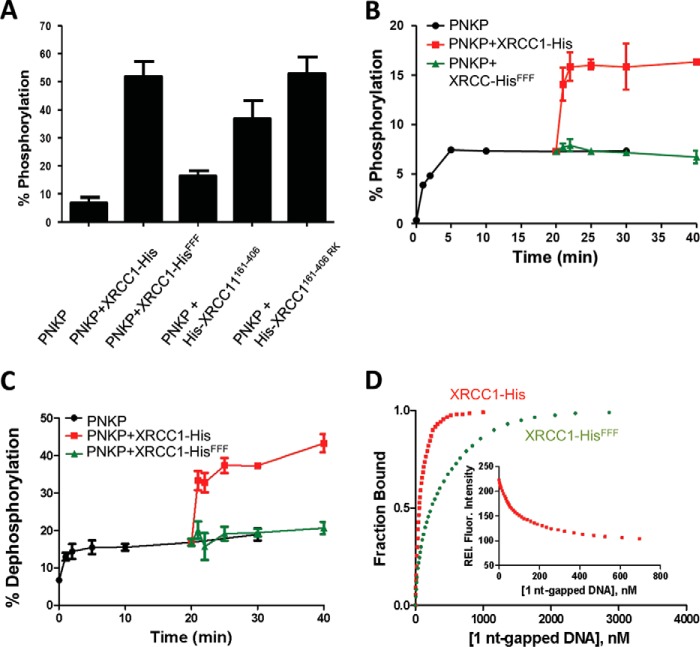
**The conserved XRCC1 phenylalanine motif is required for the phosphorylation-independent stimulation of PNKP activity.** *A*, stimulation of PNKP DNA kinase activity. 0.5 μm PNKP was incubated in the presence of [γ-^32^P]ATP with 10 μm oligonucleotide substrate and 4 μm XRCC1-His, XRCC1-His^FFF^, His-XRCC1^161–406^, or His-XRCC1^161–406-RK^ for 2 min at 37 °C. The amount of radiolabeled 5′-phosphorylated 24-mer oligonucleotide was then quantified by gel electrophoresis and autoradiography. Data are the mean ± S.D. of three independent experiments. *B*, stimulation of PNKP DNA kinase enzyme-product turnover. DNA kinase reactions (50 μl) containing 2 μm 1-nt gapped oligonucleotide substrate and 0.2 μm PNKP were conducted as above in the absence of XRCC1 for 20 min and XRCC1-His or XRCC1-His^FFF^ then added to 0.8 μm for a further 20 min. Phosphorylated oligonucleotide product was quantified at the indicated times, as above. Data are the mean ± S.D. of three independent experiments. *C*, stimulation of PNKP DNA phosphatase activity. DNA phosphatase reactions (30 μl) containing 0.33 μm 1-nt gapped oligonucleotide substrate and 0.86 μm PNKP were incubated in the absence of any XRCC1 protein for 20 min and then, where indicated, in the additional presence of 1.65 μm XRCC1-His or XRCC1-His^FFF^ for a further 20 min. 3′-Dephosphorylated oligonucleotide product was quantified at the indicated times, as above. Data are the mean ± S.D. of three independent experiments. *D*, Interaction of XRCC1-His (■) and XRCC1-His^FFF^ (●) with 1-nt gapped DNA. Proteins (30 nm) were excited at 295 nm and the fluorescence intensity at 340 nm was monitored as a function of added 1 nt-gapped DNA substrate (see inset for data with XRCC1-His). The fraction bound, *i.e.* relative fluorescence (*Rel. Fluor*.), *versus* ligand concentration is plotted.

XRCC1 stimulates PNKP activity if the latter is present at a limiting concentration (3, 4, 30, 32). We therefore examined the impact of the phenylalanine motif on PNKP activity, *in vitro* (3, 4, 32). Recombinant His-XRCC1^161–406^ stimulated the 5′-DNA kinase activity of PNKP almost to the same extent as full-length XRCC1-His, and this stimulation was ablated by mutation of the phenylalanine motif (Fig. 5*A*). This was not the case for mutations in the BRCT I domain that binds poly(ADP-ribose) (13, 14), which did not impact on PNKP stimulation (Fig. 5*A*, *His-XRCC1^161–406-RK^*). Because PNKP stimulation by XRCC1 reflects the displacement of PNKP from its 5′-phosphorylated DNA product, enabling further rounds of enzyme activity (30, 32), we next conducted single turnover experiments in which DNA substrate was present in 10-fold excess over PNKP. As expected, DNA kinase activity plateaued at ∼10% product formation, and subsequent addition of full-length XRCC1-His promoted further activity (Fig. 5*B*). In contrast, XRCC1-His^FFF^ was unable to stimulate PNKP, consistent with an inability to interact with PNKP and displace it from its 5′-phosphorylated DNA product (Fig. 5*B*). Notably, similar results were observed for DNA phosphatase activity, suggesting that the phenylalanine motif is required for stimulation of both activities of PNKP (Fig. 5*C*). Finally, because XRCC1 displaces PNKP from its DNA product in part by direct competition for DNA we also examined the influence of the phenylalanine motif on DNA binding (Fig. 5*D*). XRCC1-His bound an oligodeoxyribonucleotide duplex harboring a single-nucleotide gap ∼5-fold more tightly than did XRCC1-His^FFF^ (*K_d_* ∼ 55 and ∼250 nm, respectively), suggesting that the phenylalanine motif also influences binding to DNA.

## Discussion

**Figure 6.**
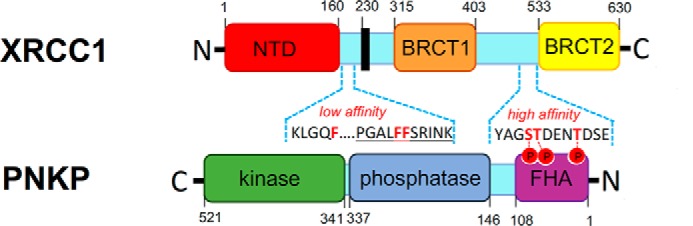
**A model for a bipartite interaction between XRCC1 and PNKP.** Shown is a schematic depicting the protein domains in XRCC1 and PNKP, with the regions of XRCC1 that are involved in the high-affinity and low-affinity interactions amplified to illustrate the protein sequence. Note that the high-affinity interaction site is mediated by the CK2 phosphorylation sites located in XRCC1 at Ser^518^/Thr^519^/Thr^523^ (residues in *red bold* and associated phosphates indicated by *red circles*) and the phosphopeptide-binding FHA domain of PNKP. The low-affinity interaction is mediated by a region spanning Phe^173^/Phe^191^/Phe^192^ (*red bold*). Note that Phe^191^/Phe^192^ form part of a putative RIR motif (*underlined*).

XRCC1 is a molecular scaffold protein that interacts with multiple components of the SSBR pathway including Pol β, PNKP, APTX, Aprataxin- and PNKP-like factor, and Lig3α (36, 37). The interaction with PNKP is likely to be particularly important, because most of the DNA strand breaks induced by oxidative damage to deoxyribose possess 3′-phosphate moieties and thus are substrates for the DNA phosphatase activity of PNKP (20, 38, 39). In addition, SSBs arising from abortive activity of topoisomerase I possess not only 3′-phosphate termini but also 5′-hydroxyl termini, which are substrates for the DNA kinase activity of PNKP. XRCC1 interacts with PNKP via a high-affinity (*K_d_*, ∼3.5 nm) phosphorylation-dependent interaction (4) and a lower affinity (*K_d_*, ∼30 nm) phosphorylation-independent interaction (4, 30, 32). Although the high-affinity interaction is well characterized, occurring via the FHA domain in PNKP and the CK2 phosphorylation sites located at Ser^518^/Thr^519^/Thr^523^ in XRCC1, the site of the low-affinity interaction is unclear. Here, we show that the low-affinity site is encoded by a highly conserved but poorly characterized region of XRCC1 containing three invariant phenylalanine residues (Phe^172^/Phe^192^/Phe^193^). Two of the three phenylalanine residues are components of a putative RIR motif; a protein interaction domain that binds Rev1 and is present in multiple translesion DNA polymerases (35). As expected, loss of the high-affinity interaction had a much greater impact on the co-immunoprecipitation of cellular PNKP by XRCC1 than did loss of the low-affinity interaction. However, loss of both interactions ablated detectable co-immunoprecipitation of cellular XRCC1 and PNKP in our experiments, suggesting that both interactions are functional. The presence of two PNKP interaction sites in XRCC1 is intriguing. We suggest a bipartite interaction model in which the high-affinity interaction acts as a molecular tether that promotes PNKP recruitment at SSBs and additionally increases the likelihood of the low-affinity interaction, which in turn stimulates PNKP directly (Fig. 6). Consistent with this, mutation of the phenylalanine motif prevented the stimulation of PNKP by XRCC1 *in vitro*. Interestingly, residue Ala^482^ has also been reported to impact the phosphorylation-independent interaction with PNKP (40). However, this residue is located close to the phosphorylation-dependent site at 518/519/523, and so it is currently unclear how this site relates to the site reported here.

The data presented here indicate that the RIR motif is functionally important for DNA strand break repair rates and cell survival following oxidative stress. However, it is currently unclear to what extent the interaction with PNKP accounts for this importance, because this motif also interacts with Rev1 (35) and with SSRP1 (41), a component of the FACT chromatin remodeling complex. Further work is required to compare directly the relative affinities of the XRCC1 RIR motif for the three binding partners, but the data available to date suggest that the affinity of XRCC1 for Rev1 *in vitro* is ∼150-fold lower than its affinity for PNKP (*K_d_* of 5 μm and 30 nm, respectively). Interestingly, in contrast to its impact on oxidative DNA damage, mutation of the RIR motif did not greatly impact the DNA base excision repair rates or survival following DNA alkylation. This is surprising because SSRP1 is implicated in the repair of such damage. This result also suggests that relatively few PNKP substrates arise during the excision repair of alkylated DNA bases, and is consistent with MMS-induced SSBs arising as products of AP endonuclease-1 activity, which does not create SSB termini that are substrates for PNKP.

In summary, we have identified within XRCC1 the site of a phosphorylation-independent interaction with PNKP and demonstrated the importance of this site for PNKP stimulation and for normal rates of SSBR and cell survival following oxidative stress. We propose that whereas the high-affinity interaction acts as a molecular tether to ensure XRCC1 and PNKP are in close proximity at SSBs arising globally across the genome, the lower-affinity interaction is important to stimulate PNKP activity, directly.

## Experimental procedures

### DNA expression constructs

The mammalian expression constructs pCD2E (empty vector) (42), pCD2E-PNK (encoding full-length PNKP) (4, 18), and pCD2E-XH (encoding full-length XRCC1-His) (11) have been described previously. pCD2E-XH^FFF^ (encoding full-length XRCC1-His^F173A/F191A/F192A^ in which the low-affinity PNKP-binding site is mutated) was created by using a QuikChange site-directed mutagenesis kit (Agilent Technologies). The bacterial expression constructs pET16b-PNK (encoding full-length N-terminally tagged His-PNKP) (23), pET16b-XH (encoding full-length XRCC1-His) (11), pTWO-E-His-XRCC1^161–406^ (encoding the truncated XRCC1 protein His-XRCC1^161–533^) (14), and pTWO-E-His-XRCC1^161–406-RK^ (encoding the truncated XRCC1 protein His-XRCC1^161–533 R335A/K369A^ in which the poly(ADP-ribose)-binding domain is mutated) (14) have been described previously. pET16b-HX^FFF^ (encoding full-length His-XRCC1^F173A/F191A/F192A^ in which the low-affinity PNKP-binding site is mutated) was created by PCR, subcloning into pCR2.1-TOPO, and finally subcloning into the NdeI site of pET16b (Novagen). All subcloned sequences were verified by Sanger sequencing.

### Recombinant proteins

His-PNKP was expressed from pET16b-PNK, purified, and labeled with AC as previously described (23, 32). Histidine-tagged XRCC1 proteins were expressed from the bacterial expression constructs indicated above and purified as previously described (43).

### Clonogenic survival assays

500 cells each cell type were plated in duplicate in 10-cm dishes and incubated for 4 h at 37○C. Cells were rinsed with PBS and either mock treated or treated with H_2_O_2_ (diluted in PBS at the indicated concentration immediately prior to use) or MMS (diluted in complete medium at the indicated concentration immediately prior to use) for 15 min at room temperature (H_2_O_2_) or 37 °C (MMS). After treatment, cells were washed twice with PBS and incubated for 10–14 days in drug-free medium at 37 °C to allow formation of macroscopic colonies. Colonies were fixed in ethanol (95%), stained with 1% methylene blue in 70% ethanol, and colonies of >50 cells were counted. Percentage survival was calculated for each drug concentration using the equation: 100 × [average mean colony number (treated plate)/average mean colony number (untreated plate)].

### Alkaline single cell-agarose gel electrophoresis (alkaline comet assay)

Subconfluent cell monolayers were trypsinized, diluted to 2 × 10^5^ cells/ml in ice-cold PBS (for H_2_O_2_ treatment) or complete media (for MMS treatment) immediately prior to treatment, and either mock-treated or treated with 150 μm H_2_O_2_ (diluted in ice-cold PBS immediately prior to use) for 20 min on ice or with the indicated concentration of MMS (diluted in complete medium) for 15 min at 37 °C. Cells were then rinsed in ice-cold PBS and incubated, where appropriate, in fresh drug-free media for the desired repair period at 37 °C. Cells (100 per data point) were then analyzed by alkaline comet assay as previously described (44) using Comet Assay IV software (Perceptive Instruments).

### Steady-state fluorescence assays

Steady-state fluorescence spectra were measured at 25 °C on a PerkinElmer Life Sciences LS-55 spectrofluorometer using 5-nm spectral resolution for excitation and emission using 10–100 nm acrylodan-labeled PNKP protein solutions as described in our earlier studies (32). In DNA binding experiments, PNKP was excited at 295 nm and changes in fluorescence were monitored at the emission maximum (340 nm). In the case of 6-acryloyl-2-dimethylaminonaphthalene-labeled PNKP protein (referred to as PNKP^WFX402-AC^ in which all the Trp except Trp^402^ located near the DNA-binding site have been replaced by Phe), excitation was at 380 nm, and the changes in AC fluorescence at the emission maximum (490 nm) were monitored. Quantitative data for the binding of DNA ligands to PNKP were obtained by measuring the quenching of the intrinsic Trp fluorescence of the protein at 340 nm following excitation at 295 nm as a function of DNA concentration. Fluorescence data were analyzed using GraphPad Prism software, as described previously (43).

### DNA kinase assays

PNKP (10 pmol) was premixed with 40 pmol of full-length XRCC1-His, His-XRCC1^FFF^, His-XRCC1^161–406^, or His-XRCC1^161–406 R335A/K369A^ at 37 °C for 5 min and then the mixtures were added to 20-μl (total volume) reactions containing kinase buffer (80 mm succinic acid, pH 5.5, 10 mm MgCl_2_, and 1 mm dithiothreitol), 0.2 nmol of 24-mer 5′-DNA kinase substrate (Integrated DNA Technologies; the single- and double-stranded DNA substrates used in this study have been described previously (32)) and 3.3 pmol of [γ-^32^P]ATP (PerkinElmer Life Sciences) and incubated for 2 min at 37 °C. 4-μl aliquots were mixed with 2 μl of 3× sequencing gel loading dye (Fisher), boiled for 10 min, and fractionated on a 12% polyacrylamide, 7 m urea sequencing gel at 200 V. Gels were imaged on a Typhoon 9400 variable mode imager (GE Healthcare, Bucks, UK) and quantified using ImageQuant 5.2 software (GE Healthcare).

### PNKP-product turnover assays

The stimulation of PNKP DNA kinase-product turnover by XRCC1 was measured essentially as described (32). Briefly, 3 × 50-μl reactions containing kinase buffer (80 mm succinic acid, pH 5.5, 10 mm MgCl_2_, and 1 mm dithiothreitol), 0.1 nmol of 1-nt gapped DNA substrate (Integrated DNA Technologies; see above), 0.2 nmol of unlabeled ATP, 3.3 pmol of [γ-^32^P]ATP (PerkinElmer Life Sciences), and 10 pmol of PNKP were incubated at 37 °C. From one of the reaction mixtures 4-μl samples were taken at 0, 1, 2, 5, 10, 20, and 30 min. To the other reaction mixtures 40 pmol of full-length wild-type XRCC1-His or His-XRCC1^FFF^ was added after 20 min incubation and 4-μl samples taken after an additional 1, 2, 5, 10, 20, and 30 min. The samples were mixed with 2 μl of 3× sequencing gel loading dye (Fisher), boiled for 10 min, and fractionated on a 12% polyacrylamide, 7 m urea sequencing gel at 200 V. Gels were scanned on a Typhoon 9400 variable mode imager and the resulting bands were quantified using ImageQuant 5.2 software.

The stimulation of PNKP DNA phosphatase-product turnover by XRCC1 was measured as follows. To prepare the 3′-DNA phosphatase substrate, 40 pmol of 1-nt gapped 45-mer duplex substrate (32) harboring a 20-mer with a 3′-phosphate terminus at the gap was radiolabeled at the 5′-terminus in a 50-μl reaction with T4 PNK (3′-phosphatase free)(New England Biolabs) in the presence of [γ-^32^P]ATP (Perkin Elmer Life Sciences). The T4 PNK was then heat inactivated and the substrate re-annealed by cooling. Each of 3 × 12-μl aliquots of radiolabeled substrate (each containing ∼10 pmol of substrate) was incubated with 16 fmol of PNKP in a 30-μl total volume in 1× T4 PNK buffer (New England Biolabs; 70 mm Tris, pH 7.6, 10 mm MgCl_2_, and 5 mm DTT) at 37 °C. 4-μl samples were taken from one of the three parallel reactions at 0, 1, 2, 5, 10, 20, and 30 min. To the other two reactions, 50 pmol of either XRCC1-His or His-XRCC1^FFF^ was added after 20 min and 4-μl samples were taken after a further 1-, 2-, 5-, 10-, and 20-min incubation. Samples were mixed with 2 μl of 3× sequencing gel loading dye (Fisher), boiled for 10 min, and fractionated on a 12% polyacrylamide sequencing gel containing 7 m urea at 1800 V for 3 h. Gels were scanned and imaged as described above.

### Affinity purification of histidine-tagged XRCC1 protein complexes

Histidine-tagged XRCC1 protein complexes were affinity purified essentially as described (18). Briefly, EM9 cells were transiently transfected with pCD2E-PNK and either empty pCD2E or the indicated pcD2E-XRCC1 expression construct and after selection in G418 for 4 days re-suspended in lysis buffer (25 mm HEPES, pH 8.0, 325 mm sodium chloride, 0.5% Triton X-100, 10% glycerol, 1 mm dithiothreitol, 25 mm imidazole, 1/100 dilution of mammalian protease inhibitor mixture (Sigma)) at a density of 1.25 × 10^6^ cells/ml and incubated on ice for 20 min. High-molecular-weight DNA was sheered by two short bursts (5 s) of sonication and the cell extracts were clarified by centrifugation. XRCC1-His complexes were purified by metal-chelate affinity chromatography by incubation of cell extract (0.3 ml) with 0.1 ml (0.05 ml bed volume) of nickel-nitrilotriacetic acid-agarose (Qiagen) for 20 min on ice with frequent mixing. The agarose beads were pelleted by gentle centrifugation at 4 °C in a microcentrifuge (3000 rpm), unbound material was removed, and the pellets were washed five times with lysis buffer before eluting bound proteins in 0.3 ml of lysis buffer containing 250 mm imidazole.

## Author contributions

C. B., R. M., M. F., and N. H. conducted experiments and edited the manuscript. K. W. C. and M. W. conceived the project and managed their respective research groups. K. W. C. wrote the manuscript and coordinated the project. All authors reviewed the results and approved the final version of the manuscript.

## Footnotes

4 The abbreviations used are:

SSBR: single-strand break repair
Pol β: polymerase β
PNKP: polynucleotide kinase/phosphatase
Lig3α: DNA ligase 3α
PARP: poly(ADP-ribose) polymerase
AOA4: ataxia oculomotor apraxia 4
FHA: forkhead-associated
RIR: Rev1-interacting region
MMS: methyl methanesulfonate
AC: 6-acryloyl-2-dimethylaminonaphthalene
nt: nucleotide.
